# AMBULATION RECOVERY PREDICTION AFTER HIP FRACTURE SURGERY USING THE HIP FRACTURE SHORT-TERM AMBULATION PREDICTION TOOL

**DOI:** 10.2340/jrm.v56.40780

**Published:** 2024-10-30

**Authors:** Nath ADULKASEM, Pojchong CHOTIYARNWONG, Ekasame VANITCHAROENKUL, Aasis UNNANUNTANA

**Affiliations:** Department of Orthopaedic Surgery, Faculty of Medicine Siriraj Hospital, Mahidol University, Bangkok, Thailand

**Keywords:** ambulation recovery, fragility hip fracture, Hip-SAP, prediction model, rehabilitation

## Abstract

**Objective:**

To develop models for predicting postoperative ambulation recovery at 3 months following fragility hip fracture surgery.

**Design:**

Cross-sectional study.

**Subjects:**

Fragility hip fracture patients aged ≥ 50 years who underwent operative treatment and completed a 3-month follow-up.

**Methods:**

Potential predictors were collected from eligible patients, while ambulation at 3 months after injury was assessed using the modified functional ambulation classification. These factors were used to develop the Hip Fracture Short-Term Ambulation Prediction, consisting of 2 models: Model 1 for postoperative ambulation and Model 2 for preinjury status recovery.

**Results:**

Among the 275 patients, 55 (20.0%) achieved good ambulation, and 59 (21.5%) returned to their preinjury status at 3 months. Age, preinjury ambulatory status, and discharge ambulatory status were identified as significant predictors of 3-month postoperative ambulation. The tool presented (Models 1 and 2) showed strong performance (area under the curve of 0.86 and 0.85, respectively) and good internal validity.

**Conclusions:**

Age, preinjury ambulatory status, and discharge ambulatory status significantly predict postoperative ambulation and preinjury status recovery at 3 months after fragility hip fracture surgery. The tool presented may aid clinicians in identifying patients who could benefit from targeted rehabilitation interventions during this crucial period.

## LAY ABSTRACT

We developed a tool to predict the walking ability of patients 3 months after hip fracture surgery. Our study included 275 patients aged 50 years and older who underwent surgery for fragility hip fractures. The tool, named Hip Fracture Short-Term Ambulation Prediction, has 2 parts: 1 forecasts walking ability after surgery, whereas the other predicts whether patients will return to their pre-injury walking level. After 3 months, 20% of the patients could walk well, and 21.5% regained their pre-injury walking ability. The most important factors influencing recovery were age, pre-injury walking ability, and walking ability at hospital discharge. Our tool accurately predicts these outcomes and may help identify patients who need extra rehabilitation support during their recovery period.

Restoring preinjury ambulatory status is a primary goal of fragility hip fracture treatment (1). However, nearly 50% of patients experience ambulation decline, which negatively impacts function and quality of life (2). To improve outcomes, current research prioritizes personalized rehabilitation tailored to postoperative ambulation capabilities (3, 4). Patients at high risk of poor postoperative ambulation may benefit from intensive rehabilitation and increased caregiver support. Therefore, accurate, individualized prognostic predictions are crucial for this personalized approach (5). Consequently, prediction models offering tailored prognoses have become valuable tools in optimizing rehabilitation protocols for fragility hip fracture recovery (6).

Most older adults with fragility hip fractures achieve peak functional recovery within the first 3 months (7–9). For example, a study by Koudouna et al. reported the greatest improvement in the Harris Hip Score between 6 and 12 weeks after surgery. Improvements continued at a slower pace between 3 and 6 months, with no significant gains observed from 6 to 12 months (8). These findings suggest that the initial 3-month window offers the most promising timeframe to optimize ambulation recovery in this patient population. Additionally, a systematic review and meta-analysis indicated that individualized, intensive exercise programmes initiated within 3 months postoperatively are most effective (10). Therefore, clinicians can potentially tailor rehabilitation intensity based on a patient’s predicted ambulation status at 3 months. This timeframe represents a critical window for maximizing functional recovery. Consequently, accurate prediction of 3-month ambulation outcomes is essential for identifying patients who may benefit from more intensive rehabilitation interventions.

Despite the development of various prediction tools, few currently offer definitive, accurate ambulation prognoses for fragility hip fracture patients at the 3-month mark (11, 12). This study aimed to address this gap by developing a prediction tool utilizing multiple potential predictors to identify patients at risk of poor postoperative ambulation at 3 months. We anticipate that accurate ambulation prediction within this critical postoperative period will empower clinicians to personalize rehabilitation programmes and home-care protocols, ultimately optimizing patient outcomes.

## METHODS

This cross-sectional study included patients aged 50 years or older who underwent operative treatment for fragility hip fractures at a single university-affiliated tertiary care centre. The procedures were conducted between July 2022 and December 2023, with all patients completing a 3-month follow-up. The exclusion criteria were multiple injuries/fractures, malignancy-related pathological fractures, recent lower extremity surgery, subsequent conditions affecting ambulation recovery, or death within 3 months post-surgery. All patients underwent early surgical treatment within 48 h. Postoperative rehabilitation was initiated within 24 h after surgery. Weight-bearing as tolerated with a walker was permitted for all participants. The Institutional Review Board approved the study protocol, questionnaire, and consent forms. This study was preregistered with the Thai Clinical Trials Registry. All participants provided informed consent, and confidentiality was maintained. The study design and reporting adhered to the STROBE statement.

We selected potential predictors based on clinical relevance and established associations with postoperative ambulation recovery in fragility hip fracture patients. These predictors were age, preinjury ambulatory status, preoperative haemoglobin, and albumin, American Society of Anesthesiologists (ASA) physical status class, body mass index, fracture type, time to surgery, hospitalization duration, and discharge ambulation status (13–15). These demographic and preoperative data were retrospectively obtained from the institute’s Fracture Liaison Service (FLS) registry.

### Outcome measurement

All patients were scheduled for a follow-up appointment 3 months after hip fracture surgery. For patients who missed this appointment, a telephone interview was conducted with the patient or their relatives to gather information on the patient’s ambulation status. We assessed patient ambulation using the Modified Functional Ambulation Classification (MFAC) (16). This validated tool classifies hip fracture patients’ ambulatory status into 7 categories (17). These categories are (I) Lyer (unable to sit without support), (II) Sitter, (III) Dependent walker (requiring firm continuous weight support), (IV) Assisted walker (requiring continuous balance or coordination support), (V) Supervised walker (requiring only verbal supervision), (VI) Indoor walker, and (VII) Outdoor walker.

At 3 months post-surgery, this study primarily assessed 2 outcomes: the patients’ ability to regain their pre-injury ambulatory status and their ability to achieve good postoperative ambulation (MFAC level VI or VII).

### Prediction model development

Following the TRIPOD guidelines (18), we developed the Hip Fracture Short-Term Ambulation Prediction (Hip-SAP) tool. It comprises 2 models: Model 1 predicts return to pre-injury ambulation, and Model 2 predicts good postoperative ambulation (MFAC VI or VII) at 3 months.

Potential predictors were modelled using multivariable logistic regression (the full model). The multivariable fractional polynomial algorithm was employed to optimize model compatibility by accommodating nonlinear variable relationships. A stepwise backward elimination algorithm was subsequently applied to achieve the best-performing model with the fewest predictors (the final model).

### Model performance and validation

Model performance for both models within the Hip-SAP tool was assessed using the area under the receiver operating characteristic curve (AUC). Model calibration, reflecting the agreement between the predicted and observed risks, was evaluated using the Hosmer–Lemeshow goodness-of-fit statistic and a calibration plot. Internal validity was assessed using bootstrap resampling. Additionally, we calculated the calibration slope, expected vs observed ratio, and calibration-in-the-large statistics to address overfitting.

### Statistical analysis and sample size

Statistical analyses were performed using Stata Statistical Software, release 18 (StataCorp LLC, College Station, TX, USA). A *p-*value < 0.05 indicated statistical significance. We used the Shapiro–Wilk test to assess data normality. The descriptive statistics included means and standard deviations for normally distributed continuous data and medians and interquartile ranges for nonnormally distributed data. Hypothesis testing for continuous data was conducted using independent *t*-tests or Mann–Whitney *U* tests, as appropriate. Categorical data were analysed using Fisher’s exact probability test and are presented as counts and percentages.

Based on the 10 events-per-variable guidelines, our sample size calculation considered 11 potential predictors and an estimated 40% return to the preinjury ambulation rate (1). The calculation indicated that 275 fragility hip fracture patients were needed for robust multivariable logistic regression analysis (19).

## RESULTS

**Fig. 1:**
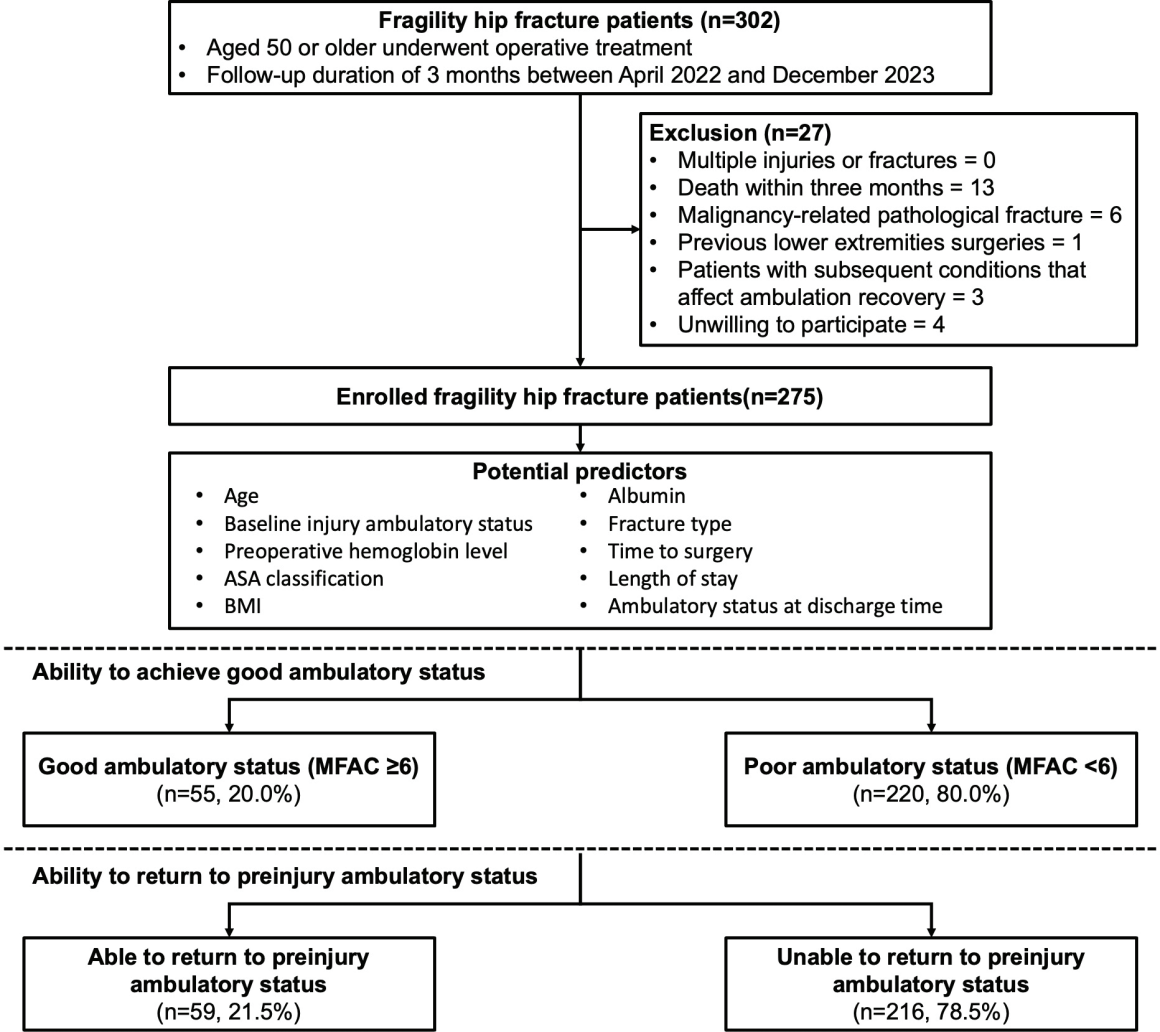
Study participant flow diagram.

Of 302 eligible fragility hip fracture patients, 275 participants were included after exclusion (Fig. 1). The cohort had a mean age of 80.4 years (8.8) and a mean body mass index of 22.4 kg/m^2^ (3.8), and 77% of the patients were female. The mean preinjury MFAC score was approximately 5.8, with 55% of patients having femoral neck fractures. The mean time to surgery was approximately 2 days, with a total hospital stay of 13 days. At discharge, 67.3% could walk with firm weight support (MFAC III).

At 3 months postoperatively, 55 individuals (20.0%) achieved an MFAC ≥ 6, and 59 (21.5%) regained their preinjury ambulatory status. Thirty patients (10.1%) achieved both of these outcomes. Age and preinjury ambulatory level were significantly associated with both outcomes. Additionally, fracture type, albumin level, length of hospital stays, and discharge ambulatory level were associated with good postoperative MFAC (Tables I–II).

**Table I:** Demographic characteristics by postoperative ambulation status (modified functional ambulation classification [MFAC] ≥ 6) at 3 months

Demographic data	Total *n* = 275	MFAC ≥ 6 (*n* = 55, 20.0%)	MFAC < 6 (*n* = 220, 80.0%)	*p*-value
Age (years), mean (SD)	80.4 (8.8)	74.4 (9.3)	81.9 (8.1)	< 0.001
Female, *n* (%)	212 (77.1%)	39 (70.9%)	173 (78.6%)	0.281
BMI (kg/m^2^), mean (SD)	22.4 (3.8)	22.2 (3.5)	22.4 (3.9)	0.786
Preinjury MFAC, mean (SD)	5.8 (1.5)	6.8 (0.7)	5.5 (1.5)	< 0.001
Preinjury MFAC ≥ 6, *n* (%)	195 (70.9%)	53 (96.4%)	142 (54.6%)	< 0.001
Preinjury MFAC < 6, *n* (%)	80 (29.15)	2 (3.6%)	78 (35.5%)	
Fracture, *n* (%)				0.010
Femoral neck fracture	152 (55.3%)	39 (70.9%)	113 (51.4%)	
Intertrochanteric fracture	123 (44.7%)	16 (29.1%)	107 (48.6%)	
Preoperative Hb (g/dL), mean (SD)	11.1 (1.7)	11.2 (1.8)	11.1 (1.7)	0.723
Albumin level (g/dL), mean (SD)	3.1 (3.9)	4.4 (8.7)	2.8 (0.4)	0.006
ASA level, median (IQR)	3 (2, 3)	2 (2, 3)	3 (2, 3)	0.090
Time to surgery (days), mean (SD)	2.1 (1.8)	1.9 (1.3)	2.1 (1.9)	0.424
Length of stay (days), mean (SD)	12.5 (7.9)	10.1 (7.9)	13.1 (7.8)	0.013
MFAC at discharge time, mean (SD)	2.6 (0.6)	3.0 (0.5)	2.5 (0.7)	< 0.001
MFAC at discharge time ≥ 3, *n* (%)	185 (67.3%)	52 (94.6%)	133 (60.5%)	< 0.001
MFAC at discharge time < 3, *n* (%)	90 (32.7%)	3 (5.5%)	87 (40.0%)	

BMI: body mass index; Hb: haemoglobin; ASA: American Society of Anesthesiologists physical status classification.

**Table II:** Demographic characteristics by ability to return to pre-injury ambulation status at 3 months

Demographic data	Total *n* = 275	Able to return (*n* = 59, 21.5%)	Unable to return (*n* = 216, 78.5%)	*p*-value
Age, years, mean (SD)	80.4 (8.8)	76.6 (9.2)	81.5 (8.5)	< 0.001
Female, *n* (%)	212 (77.1%)	45 (76.3%)	167 (77.3%)	0.862
Body mass index (kg/m^2^), mean (SD)	22.4 (3.8)	22.9 (4.2)	22.2 (3.7)	0.206
Preinjury MFAC, mean (SD)	5.8 (1.5)	5.0 (1.9)	6.0 (1.2)	0.001
Preinjury MFAC ≥ 6, *n* (%)	195 (70.9%)	28 (47.5%)	167 (77.3%)	
Preinjury MFAC < 6, *n* (%)	80 (29.1%)	31 (52.5%)	49 (22.7%)	
Fracture, *n* (%)				1.000
Femoral neck fracture	152 (55.3%)	33 (55.9%)	119 (55.1%)	
Intertrochanteric fracture	123 (44.7%)	26 (44.1%)	97 (44.9%)	
Preoperative Hb (g/dL), mean (SD)	11.1 (1.7)	11.1 (1.9)	11.1 (1.7)	0.807
Albumin level (g/dL), mean (SD)	3.1 (3.9)	2.9 (0.4)	3.2 (4.4)	0.598
ASA level, median (IQR)	3 (2, 3)	3 (2, 3)	3 (2, 3)	0.729
Time to surgery (days), mean (SD)	2.1 (1.8)	1.8 (0.9)	2.2 (2.0)	0.145
Length of stay (days), mean (SD)	12.5 (7.9)	11.1 (7.5)	12.9 (8.0)	0.136
MFAC at discharge time, mean (SD)	2.6 (0.6)	2.8 (0.6)	2.6 (0.7)	0.056
MFAC at discharge time ≥ 3, *n* (%)	185 (67.3%)	42 (71.2%)	143 (66.2%)	< 0.001
MFAC at discharge time < 3, *n* (%)	90 (32.7%)	17 (28.8%)	73 (33.8%)	0.533

MFAC: Modified Functional Ambulation Classification; Hb: haemoglobin; ASA: American Society of Anesthesiologists physical status classification.

Multivariable logistic regression with the multivariable fractional polynomial algorithm revealed that age, preinjury MFAC, and discharge MFAC significantly predicted good ambulation and a return to preinjury status at 3 months (Tables III–IV). The preoperative haemoglobin level, although initially significant, was excluded during backward elimination. Age, preinjury MFAC, and discharge MFAC were thus incorporated into both Model 1 and Model 2.

**Table III:** Model 1: multivariable logistic regression of predictors for good postoperative ambulation (MFAC ≥6) at 3 months

Demographic data	Covariate transformation			Full model analysis					Final model 2				
	Association	df	Formula	OR	β	95%	CI	*p*-value	OR	β	95%	CI	*p*-value
Age, years	Linear	1	Age–80.4	0.92	–0.08	–0.13	–0.03	0.001	0.93	–0.08	–0.12	–0.03	< 0.001
Female	Linear	1		0.66	–0.42	–1.30	0.47	0.356					
Body mass index (kg/m^2^)	Linear	1	BMI–22.4	1.01	0.01	–0.09	0.11	0.818					
Preinjury MFAC (PreMFAC)	Nonlinear	2	PreMFA–194.7	1.01	0.01	0.01	0.02	0.000	1.01	0.01	0.01	0.02	< 0.001
Fracture													
Femoral neck	Linear	1		0.96	–0.04	–0.91	0.83	0.930					
Intertrochanteric	Ref.												
Preoperative Hb (g/dL)	Linear	1	Hb–11.1	0.75	–0.29	–0.54	–0.03	0.028					
Albumin level (g/dL)	Linear	1	Alb–3.1	1.99	0.69	–0.32	1.70	0.182					
ASA level	Linear	1	ASA–2.6	1.12	0.12	–0.58	0.81	0.744					
Time to surgery (days)	Linear	1	Time to surgery–2.1	0.96	–0.04	–0.34	0.26	0.814					
Length of stay (days)	Linear	1	Length of stay–12.5	0.96	–0.04	–0.11	0.02	0.161					
MFAC at discharge time (DcMFAC)	Linear	1	DcMFAC–2.6	4.58	1.52	0.49	2.56	0.004	4.18	1.43	0.50	2.36	0.003
Intercept					–2.65	–3.82	–1.49		0.05	–2.93	–3.77	–2.10	

MFAC: Modified Functional Ambulation Classification; Hb: haemoglobin; ASA: American Society of Anesthesiologists physical status classification.

**Table IV:** Model 2: multivariable logistic regression of predictors for return to pre-injury ambulatory status at 3 months

Demographic data	Covariate transformation			Full model analysis					Final model 2				
	Association	df	Formula	OR	β	95%	CI	*p*-value	OR	β	95%	CI	*p*-value
Age, years	Linear	1	Age–80.4	0.91	–0.09	–0.14	–0.05	< 0.001	0.92	–0.08	–0.13	–0.04	< 0.001
Female	Linear	1		0.86	–0.16	–1.02	0.71	0.726					
Body mass index (kg/m^2^)	Linear	1	BMI–22.4	1.07	0.07	–0.02	0.17	0.146					
Preinjury MFAC (PreMFAC)	Nonlinear	4	PreMFAC–194.7	0.74	–0.30	–0.41	–0.19	< 0.001	0.76	–0.27	–0.38	–0.17	< 0.001
			PreMFAC*ln(PreMFAC)–342.2	1.15	0.14	0.09	0.19	< 0.001	1.14	0.13	0.08	0.18	< 0.001
Fracture													
Femoral neck	Linear	1		0.92	–0.08	–0.91	0.75	0.848					
Intertrochanteric	Ref.												
Preoperative Hb (g/dL)	Linear	1	Hb–11.1	0.87	–0.14	–0.36	0.08	0.223					
Albumin level (g/dL)	Linear	1	Alb–3.1	0.88	–0.13	–0.53	0.26	0.512					
ASA level	Linear	1	ASA–2.6	1.08	0.08	–0.57	0.73	0.809					
Time to surgery (days)	Linear	1	Time to surgery–2.1	0.86	–0.15	–0.50	0.20	0.399					
Length of stay (days)	Linear	1	Length of stay–12.5	0.96	–0.04	–0.10	0.01	0.138					
MFAC at discharge time (DcMFAC)	Linear	1	DcMFAC–2.6	3.39	1.22	0.44	2.01	0.002	3.04	1.11	0.37	1.85	0.003
Intercept					–4.10	–5.51	–2.68		0.02	–3.90	–5.07	–2.74	

MFAC: Modified Functional Ambulation Classification; Hb: haemoglobin; ASA: American Society of Anesthesiologists physical status. classification.

**Fig. 2:**
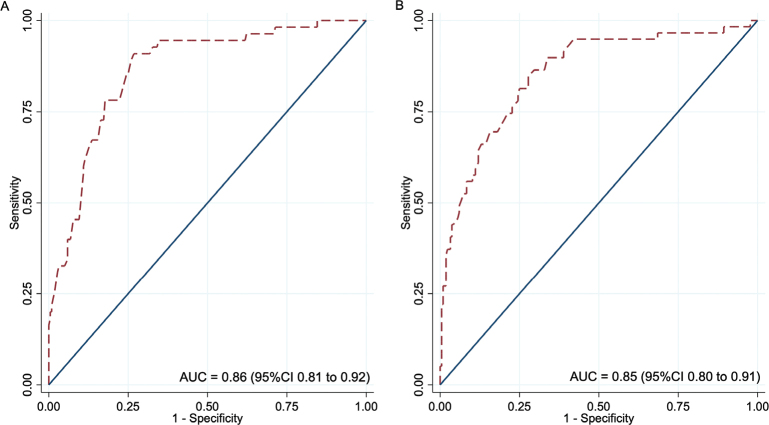
Receiver operating characteristic curves for postoperative ambulation prediction models. (A) Model 1: Predicts good ambulatory status at 3 months. (B) Model 2: Predicts return to preinjury ambulatory status at 3 months.

**Fig. 3:**
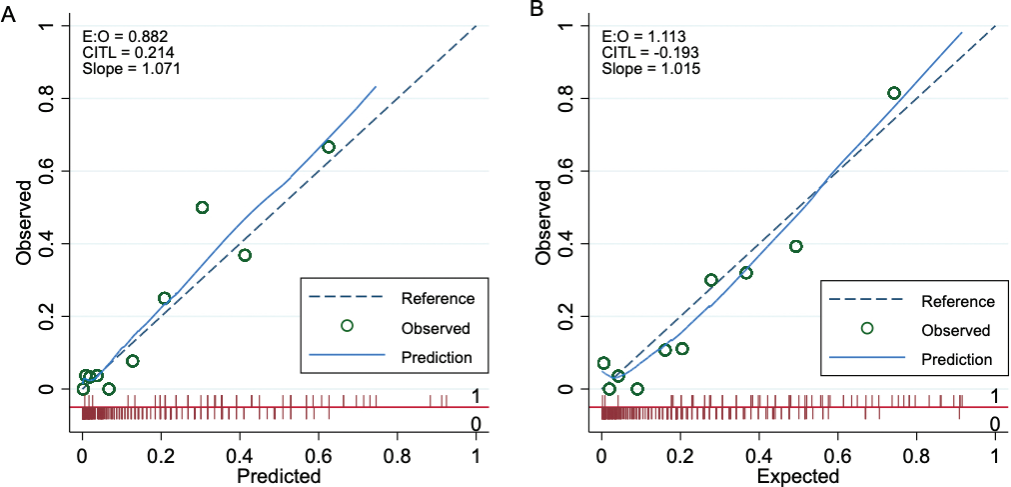
Calibration plots for postoperative ambulation prediction models. (A) Prediction of good ambulatory status at 3 months. (B) Prediction of return to preinjury ambulatory status at 3 months.

The 2 models performed well, with AUC values of 0.86 (95% CI 0.81–0.92) for Model 1 and 0.85 (95% CI 0.80–0.91) for Model 2 (Fig. 2). The calibration plots showed excellent agreement between predicted and observed probabilities. Minimal overfitting was demonstrated by expected/observed ratios of 0.88 and 1.11, calibration-in-the-large values of 0.21 and –0.19, and calibration slopes of 1.07 and 1.02 for Model 1 and Model 2, respectively (Fig. 3). Bootstrap resampling confirmed strong internal validity (Table V).

**Table V:** Bootstrap validation: assessment of prediction model optimism

Prediction model	Model c-statistics			Bootstrap c-statistics		
		95%	CI		95%	CI
1. Model 1	0.864	0.812	0.917	0.862	0.810	0.917
2. Model 2	0.854	0.797	0.911	0.852	0.795	0.912

## DISCUSSION

This study demonstrated that age, preinjury ambulatory status, and ambulatory status at discharge significantly predict postoperative ambulation at 3 months following hip fracture surgery. Our Hip-SAP tool (Models 1 and 2) accurately incorporates these factors to identify patients likely to achieve good postoperative ambulation and those who may regain their preinjury functional status. As this 3-month window represents a crucial period for functional recovery, the Hip-SAP tool offers valuable insights for clinicians. It facilitates the identification of patients who could benefit from targeted, intensive rehabilitation, ultimately optimizing their outcomes.

Few tools exist for predicting 3-month postoperative ambulation. Tomita et al. (12) introduced a model incorporating factors such as preinjury residence, dementia status, admission serum albumin, and the Barthel index measured 2 weeks post-surgery. However, while providing acceptable accuracy (AUC = 0.710), this model was specifically tailored for patients with intertrochanteric fractures undergoing cephalomedullary nailing. In contrast, the retrospective cohort study by Yamamoto et al. (11) proposed a tool that leveraged early postoperative cumulative ambulation scores on days 1, 3, and 5 alongside the preinjury Barthel index and cognitive function assessments. This instrument achieved a good AUC of 0.855, effectively distinguishing fragility hip fracture patients capable of independent ambulation at the 3-month mark (11). Building upon these foundations, our Hip-SAP tool offers 2 models: Model 1 predicts good postoperative ambulation, while Model 2 predicts the recovery of preinjury status, providing broader clinical applicability. Additionally, the Hip-SAP tool simplifies the evaluation process, requiring only age, preinjury ambulation status, and discharge ambulation level, thus facilitating assessments by clinicians.

Younger patients are more likely to achieve good ambulation and return to baseline levels within 3 months after hip fracture. While patients with greater preinjury mobility have a better chance of good post-operative ambulation, they may not fully regain their original function. This finding aligns with previous research demonstrating that 50–70% of hip fracture patients experience ambulatory decline (1, 20, 21). Our findings emphasize the strong association between discharge ambulation status and 3-month recovery, which reinforces prior studies (13, 22). This suggests that focusing inpatient rehabilitation on optimizing ambulation at discharge could significantly enhance overall postoperative recovery (9, 23, 24).

The Hip-SAP tool we developed offers predictive insights that can guide patients and their families in anticipating the expected level of ambulation 3 months after surgery. Previous research has demonstrated that effective information sharing is critical during the transition from hospital to home (25). The predictions provided by the Hip-SAP tool can assist in preparing for postsurgical care, including making necessary home modifications for accessibility, arranging caregiver support, and planning transportation (26). Additionally, we can tailor more focused care strategies by identifying patients who are unlikely to achieve good ambulation or return to their pre-injury status within 3 months. These patients may benefit from specialized rehabilitation programmes, such as inpatient-intensive rehabilitation in an intermediate care facility or a customized home-based rehabilitation programme (27, 28). These interventions have improved outcomes in selected patients with fragility hip fractures.

This investigation has several strengths. The outcome data were precisely collected cross-sectionally within 3 months after surgery, effectively minimizing recall bias. Despite the retrospective collection of predictor variables from the institute’s Fracture Liaison Service Registry, these data were initially acquired prospectively at the point of patient injury, thus reducing potential study biases. The Hip-SAP tool, which leverages only 3 straightforward predictors, offers enhanced practicality for clinical implementation.

This study has some limitations. First, the study’s initial sample size estimation was based on an anticipated 40% of hip fracture patients regaining their preinjury ambulatory status post-surgery (1). However, our findings indicated that only approximately 20% achieved this outcome, potentially affecting the ability of the multivariable logistic regression model to identify certain predictor associations. Nonetheless, our final prediction tool comprised only 3 predictors, ensuring sufficient statistical power with 30 events and validating the sample size for the Hip-SAP tool analysis. Second, not all potential factors were included in our analysis. For example, we did not record patients’ cognitive status or the time when each patient first ambulated, both of which have been shown to influence functional outcomes in hip fracture patients (29–31). Third, the Hip-SAP tool was developed to provide informative predictions without a specific cutoff probability or direct recommendations regarding which patients might benefit from specialized rehabilitation protocols. Further research is necessary to enhance the practical application of our model. Finally, the single-institute nature of the study may restrict the wider generalizability of the Hip-SAP tool. Ideally encompassing multiple centres, future research is essential for the external validation of the Hip-SAP tool, particularly in predicting short-term ambulation recovery after hip fracture surgery.

In conclusion, this study demonstrated that age, preinjury ambulatory status, and discharge ambulatory status significantly predict 3-month postoperative ambulation and return to preinjury status in fragility hip fracture patients. This 3-month window is critical for functional recovery, so the Hip-SAP tool provides clinicians with valuable insights. It facilitates the identification of patients who would likely benefit from targeted, intensive rehabilitation interventions to maximize their outcomes.

## Data Availability

The data that support the findings of this study are available from the corresponding author upon reasonable request. The data are not publicly available due to privacy or ethical restrictions.

## Study registration number

The Institutional Review Board approved the study protocol, questionnaire, and consent forms (COA no. Si 318/2022). This study was preregistered with the Thai Clinical Trials Registry (TCTR20220608002). All participants provided informed consent prior to data collection.
